# The temporal sequence of myasthenia gravis and neuromyelitis optica spectrum disorder: a case report and systematic review of 74 patients

**DOI:** 10.3389/fneur.2026.1747855

**Published:** 2026-02-13

**Authors:** Zhenyu Niu, Yi Bao, Lanqiu Yao, Jingru Ren, Jianchun Wang, Jing Guo, Nan Zhang, Feng Gao, Hongjun Hao, Siwei Chen, Ran Liu

**Affiliations:** 1 Department of Neurology, Peking University First Hospital, Beijing, China; 2 Department of Neurology, Taihe Hospital, Hubei University of Medicine, Shiyan, China; 3 Department of Population Health, New York University, New York, NY, United States

**Keywords:** acetylcholine receptor antibody, aquaporin 4, eculizumab, myasthenia gravis, neuromyelitis optica spectrum disorder

## Abstract

**Background:**

The co-occurrence of myasthenia gravis (MG) and neuromyelitis optica spectrum disorder (NMOSD) is rare, and their temporal sequence and shared pathogenesis remain poorly understood.

**Methods:**

We present the case of a 43-year-old woman with pre-existing acetylcholine receptor antibody (AChR-Ab) positive ocular MG who developed aquaporin-4 antibody (AQP4-Ab) positive NMOSD. Additionally, we conducted a systematic literature review up to July 2024 to identify similar cases.

**Results:**

Including the present case, 74 patients were analyzed. The cohort showed a marked female predominance (89.2%). MG onset significantly preceded NMOSD onset in most patients (93.2%), with a younger mean age at onset (28.19 vs. 41.02 years, *p* < 0.001) and a mean interval of 12.56 years. Among tested patients, AChR-Ab was positive in 90.5% and AQP4-Ab in 73.0%. In our case, administration of the complement C5 inhibitor eculizumab led to marked clinical improvement after failure of high-dose steroid therapy.

**Conclusion:**

This study suggests a consistent temporal pattern wherein MG predominantly precedes NMOSD, often by years, suggesting a possible shared autoimmune predisposition. The observed response to eculizumab highlights complement pathway inhibition as a potentially effective therapeutic strategy for this complex overlap syndrome, warranting further investigation.

## 1 Introduction

Myasthenia gravis (MG) and neuromyelitis optica spectrum disorder (NMOSD) are distinct antibody-mediated autoimmune neurological disorders. MG targets the neuromuscular junction, whereas NMOSD involves demyelination in the central nervous system (CNS), leading to optic neuritis and transverse myelitis (1, 2). Although MG and NMOSD are distinct clinical entities, the rare co-occurrence or sequential development of both diseases in a single individual suggests a potential shared autoimmune predisposition, leading to a level of clinical complexity and heterogeneity that surpasses what is typically seen in either condition alone. Understanding this temporal pattern and the underlying immunopathological mechanisms is crucial for improving patient management.

In this case report, we present a rare and illustrative case of a 43-year-old woman with a known history of MG who developed NMOSD, and who demonstrated a poor response to steroids but a remarkable one to eculizumab. The rapid succession of multiple severe symptoms, combined with the atypical treatment response, makes this case exceptionally uncommon in clinical practice. To better understand and contextualize this phenomenon, we conducted a comprehensive review of 73 published case reports to date (3–28). By integrating this case with a systematic review of the literature, we aim to delineate the clinical characteristics, temporal sequence, and therapeutic implications of this rare association.

## 2 Case presentation

A 43-year-old woman was admitted for a one-month history of intractable hiccups and vomiting culminating in rapid visual loss and ascending sensorimotor deficits. Her symptoms began approximately one month prior with persistent hiccups and severe, intractable vomiting. About ten days after the initial gastrointestinal symptoms, she developed generalized pruritus, neck pain, band-like sensation around the chest, and progressive bilateral lower limb numbness and weakness, which was more pronounced on the right side. This sensorimotor deficit ascended to involve her upper limbs, primarily the right arm, and was accompanied by urinary retention and constipation. In the ten days immediately preceding admission, she noted a rapid and significant deterioration of vision in her left eye.

Her medical history was significant for ocular myasthenia gravis (MG), characterized initially by left-sided ptosis and diplopia, and diagnosed in 2019 with positive anti-acetylcholine receptor antibody (AChR-Ab). She was initially treated with pyridostigmine (60 mg, tid), and received mycophenolate mofetil (500 mg, bid) three months later because she developed bilateral ptosis. She maintained stable symptom control on these medications for approximately five years. Notably, her ptosis did not recur after discontinuation of these medications one month prior due to severe vomiting. Other past medical histories included a COVID-19 infection in 2023 and herpes zoster affecting the right waist two months before admission.

On admission, neurological examination revealed severely impaired left-eye visual acuity (counting fingers at 30 cm and hand motion at 50 cm), reduced strength in neck flexor (grade 5-) and bilateral limbs (grade 5- in left limbs and grade 4 in right limbs). Additional findings included symmetrically decreased muscle tone, hyperreflexia (more prominent on the right), positive pathological reflexes, and diminished sensation to pinprick and vibration below the T4 dermatome bilaterally. No meningeal signs were detected. The Expanded Disability Status Scale (EDSS) score at admission was 4.0.

**Figure 1:**
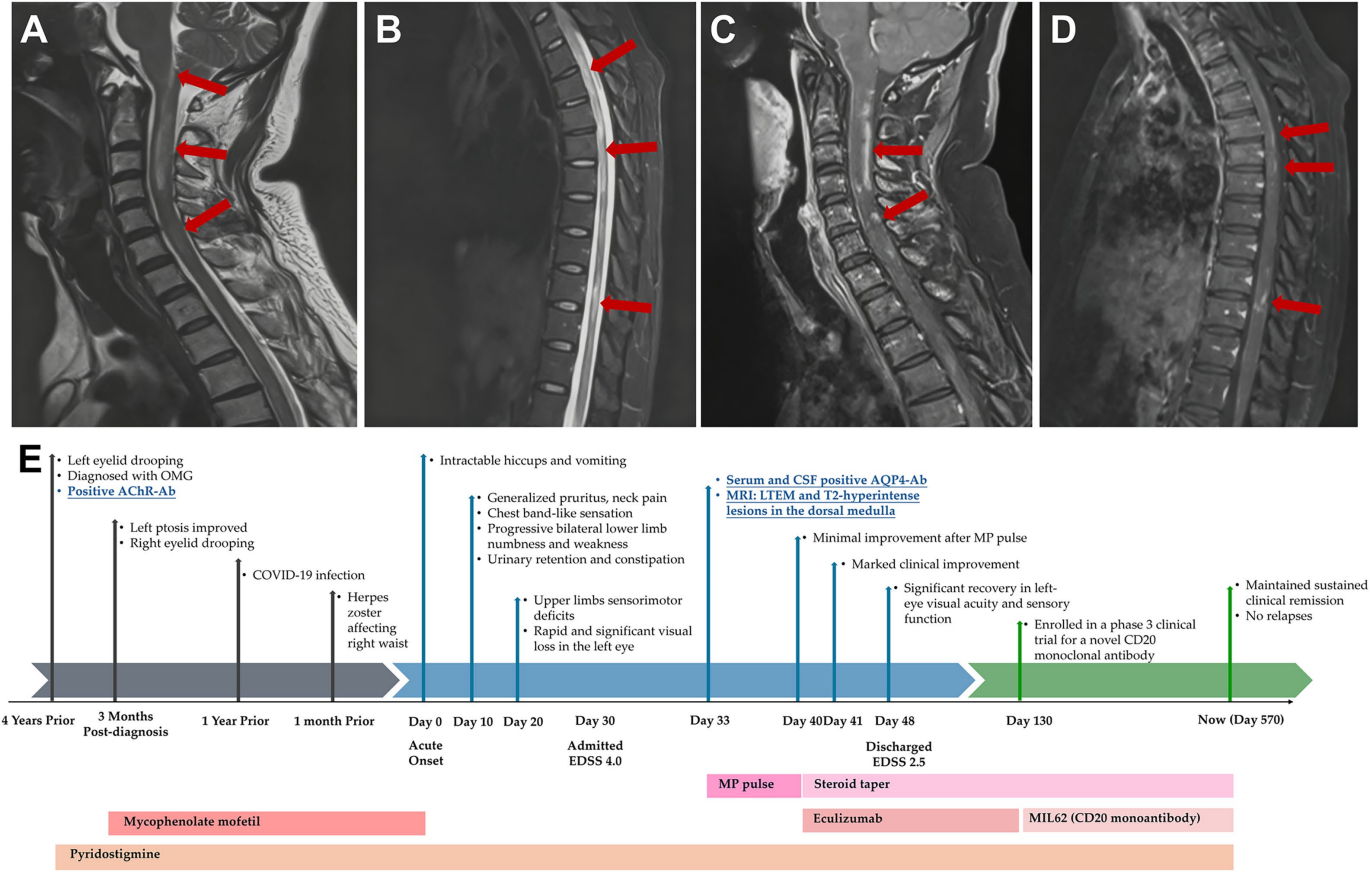
Magnetic resonance imaging and clinical timeline of the patient. **(A–D)** MRI of the patient at time of admission: sagittal view of the cervical and thoracic spine revealed multiple T2-weighted hypersignal lesions in the posterior bulbar (area postrema), cervical **(A)** and thoracic medulla **(B)**, with some lesions showing contrast enhancement in contrast-enhanced T1-weighted images (**C,D**; arrows). **(E)** Detailed graphical timeline of the patient’s symptoms. IVMP, intravenous methylprednisolone; EDSS, Expanded Disability Status Scale.

Routine blood tests, including inflammatory markers, thyroid function, and metabolic panels (homocysteine, vitamin B12, folate), were unremarkable except for hypokalemia. Due to clinical suspicion of spinal cord involvement, MRI of the brain and spinal cord was performed, which revealed a linear T2-hyperintensity in the dorsal medulla (area postrema) and longitudinally extensive transverse myelitis spanning multiple cervical and thoracic segments, with patchy gadolinium enhancement (Figures 1A–D). Regarding her myasthenia gravis (MG) history, repetitive nerve stimulation indicated postsynaptic neuromuscular junction dysfunction, and serum AChR-Ab remained positive. Tests for immunoglobulins and complement showed an elevated C1q level (242.30 mg/L). Tumor marker screening revealed mild elevation of carbohydrate antigen 72–4 (10.29 U/mL), although chest and abdominal CT scans showed no thymic pathology or other tumors. Further evaluations included visual evoked potentials, which showed an absent P100 wave in the left eye and delayed latency in the right, and bladder ultrasound demonstrating mild post-void residual urine (76 mL).

Based on imaging findings indicative of myelitis, cerebrospinal fluid (CSF) analysis was performed. CSF analysis revealed a normal cell count with mildly elevated protein (0.55 g/L). Neuroimmunological testing showed type III oligoclonal bands, an elevated albumin quotient (15.23 × 10^−3^), and positive aquaporin-4 antibody (AQP4-Ab) in both serum and CSF (titer 1:40) by cell-based assay. Testing for myelin oligodendrocyte glycoprotein antibody and paraneoplastic antibodies was negative in both serum and CSF. Based on the 2015 international diagnostic criteria, a diagnosis of NMOSD was confirmed in the context of pre-existing MG.

Following the diagnosis, the patient received high-dose intravenous methylprednisolone pulse therapy (1 g/day for 5 days), which resulted in little improvement in her visual or sensorimotor symptoms. Eculizumab was initiated 1 week later at a dose of 900 mg weekly for 4 weeks, followed by 1,200 mg every 2 weeks. Marked clinical improvement was observed within 24 h after first eculizumab infusion. After 1 week, her left-eye visual acuity had improved to counting fingers at 2 meters, sensory function had improved above the T8 level, and her EDSS score decreased to 2.5. She was discharged after the second eculizumab infusion. She received eculizumab for 3 months in total and was subsequently enrolled in a phase 3 clinical trial for a novel, domestically produced third-generation CD20 monoclonal antibody MIL62, on which she has remained relapse-free to date. A detailed timeline summarizing the clinical progression, diagnostic evaluations, and treatments is shown in Figure 1E.

## 3 Literature review

### 3.1 Search strategy and study selection

We conducted a systematic literature search without language restrictions using PubMed, Embase, Web of Science, and Google Scholar from inception to July 2024. The search strategy employed Boolean operators: (“myasthenia gravis” OR “MG”) AND (“neuromyelitis optica” OR “NMOSD” OR “NMO” OR “Devic’s disease”). Two authors (Y.B. and Z.N.) independently screened titles/abstracts and reviewed full texts of potentially eligible articles. Duplicate records were removed, and any discrepancies were resolved through discussion or by consultation with a third reviewer (S.C. or R.L.).

The study selection process followed the Preferred Reporting Items for Systematic Reviews and Meta-Analyses (PRISMA) guidelines and is summarized in a flow diagram (Supplementary Figure S1). Articles were included if they reported one or more patients with co-occurring or sequentially diagnosed MG and NMOSD. We applied this inclusive criterion to capture cases published before the widespread availability of AQP4-IgG testing or the formalization of modern NMOSD diagnostic criteria, provided their clinical features were consistent with the current NMOSD spectrum. Articles describing MG co-occurring with typical multiple sclerosis without these distinctive NMOSD features were excluded. Review articles, commentaries, and studies lacking sufficient individual clinical data were also excluded. The reference lists of all included articles were manually searched to identify additional relevant reports.

### 3.2 Statistical methods

Statistical analysis was performed using *IBM SPSS* version 20.0. Descriptive statistics included percentages, frequencies, and means. Categorical data were represented as counts (*n*) with percentage and analyzed by Fisher’s exact test, whereas continuous data were expressed as means ± standard deviation (x̅ ± s) and analyzed using the t-test. A *p*-value <0.05 was considered statistically significant.

### 3.3 Summary of findings

**Figure 2:**
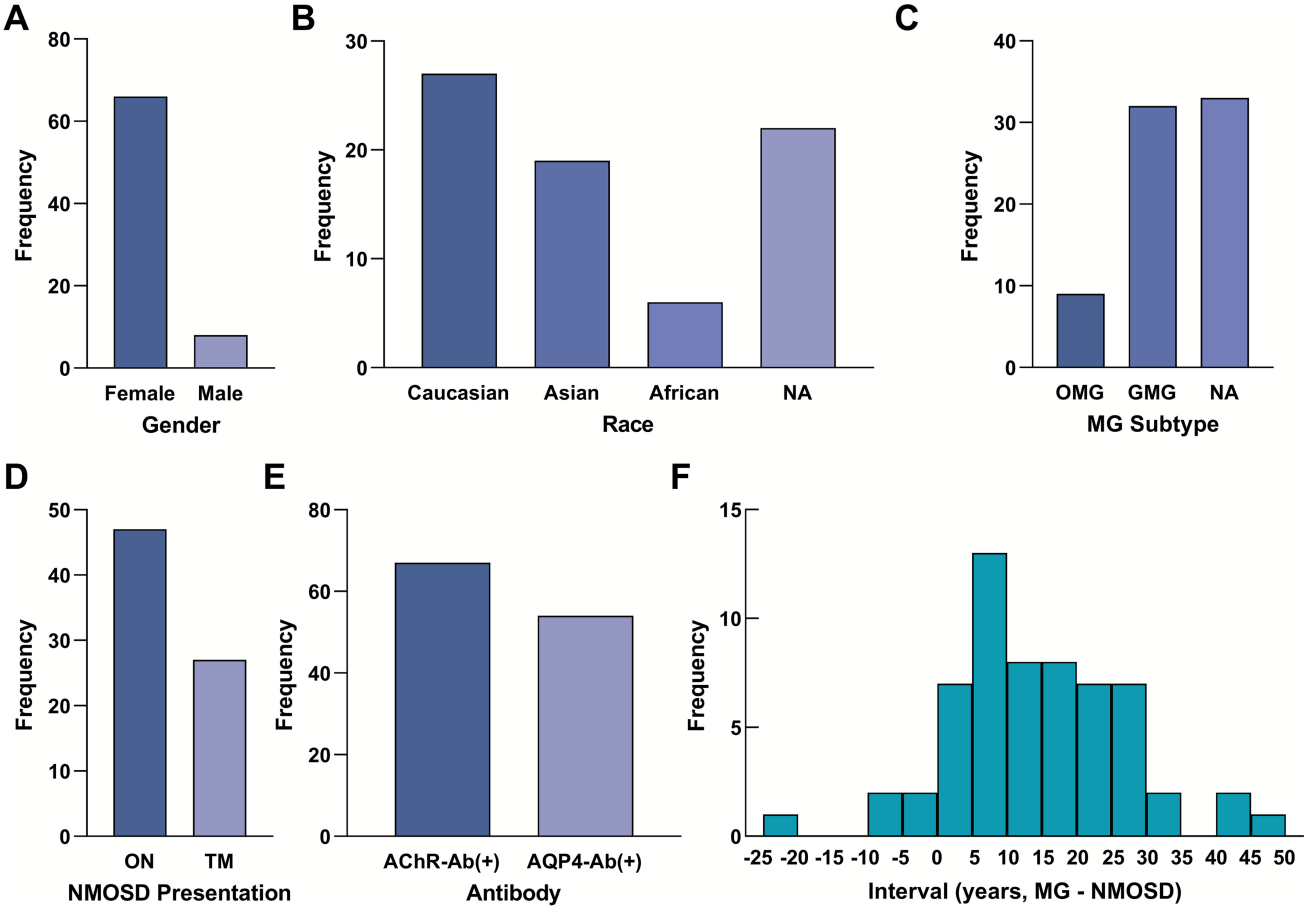
Demographic, clinical, and serological profile of 74 patients with coexisting myasthenia gravis and neuromyelitis optica spectrum disorder. **(A)** Gender distribution of the cohort (Female: 89.2%, *n* = 66; Male: 10.8%, *n* = 8). **(B)** Racial distribution (Caucasian: 36.5%, *n* = 27; Asian: 25.7%, *n* = 19; African: 8.1%, *n* = 6; Unknown: 29.7%, *n* = 22). **(C)** Distribution of MG types (Generalized: 43.2%, *n* = 32; Ocular: 12.2%, *n* = 9; Unknown: 44.6%, *n* = 33). **(D)** Initial NMOSD presentation (ON: 63.5%, *n* = 47; TM: 36.5%, *n* = 27). **(E)** Seropositivity rates for key antibodies (AChR-Ab positive: 90.5%, *n* = 67; AQP4-Ab positive: 73.0%, *n* = 54). **(F)** Histogram showing the distribution of the interval between the onset of MG and NMOSD (*n* = 57 with reported interval data). Negative values indicate that NMOSD onset preceded MG. The mean interval is 12.56 ± 12.99 years. MG, myasthenia gravis; NMOSD, neuromyelitis optica spectrum disorder; ON, optic neuritis; TM, transverse myelitis; AChR-Ab, acetylcholine receptor antibody; AQP4-Ab, aquaporin-4 antibody.

Including our present case, a total of 74 patients (73 from literature and 1 index case) with coexisting MG and NMOSD were analyzed. Detailed data are provided in Table 1, while the key demographic, clinical, and serological characteristics of this cohort are summarized in Figure 2. A pronounced female predominance was observed, with 66 female patients (89.2%) and 8 male patients (10.8%) (Figure 2A). The cohort was ethnically diverse, with the majority being of Caucasian (36.5%) or Asian (25.7%) descent (Figure 2B). Regarding the clinical subtype of MG, generalized disease was most common (43.2%), followed by ocular MG (12.2%); the subtype was not specified in 44.6% of reports (Figure 2C). The initial presentation of NMOSD was optic neuritis (ON) in 63.5% of patients and transverse myelitis (TM) in 36.5% (Figure 2D).

**Table 1:** Characteristics of patients diagnosed with the coexistence of NMOSD and MG.

Case and references	Count (*n*)	Gender	Race	MG type	Age of MG onset (years)	Age of NMOSD onset (years)	Interval (years)	AChR Antibody	AQP4 Antibody	Thymectomy	Pathology	NMOSD presentation	NMOSD treatment
Antoine et al. (3)	1	M	NA	Generalized	49	49	0.6	(+)	NA	(+)	Thymoma	TM	IVMP, PLEX, cyclophosphamide
Balarabe et al. (4)	1	F	African	Generalized	8	14	6	(+)	(+)	(+)	Hyperplasia	ON	IVMP, oral steroids
Bates et al. (5)	1	F	NA	NA	39	54	15	(+)	(+)	(+)	NA	TM	IVMP, eculizumab
Bichuetti et al. (6)	1	F	Caucasian	Generalized	27	31	4	NA	NA	(+)	NA	TM	IVMP, AZA, oral steroids
Castro-Suarez et al. (7)	2	2F	NA	Ocular	20, 18	49, 23	29, 5	2(+)	2(+)	2(+)	Hyperplasia	1ON, 1TM	IVMP, AZA, oral steroids
Etemadifar et al. (8)	1	F	Caucasian	Generalized	42	33	−9	(+)	(+)	NA	NA	ON	PLEX, mitoxantrone, AZA, oral steroids
Furukawa et al. (9)	2	2F	Asian	NA	23, 63	48, 63	25, 0	2(+)	2(−)	1(+), 1(−)	1Hyperplasia, 1NA	2ON	IVMP, oral steroids
Gotkine et al. (10)	1	F	NA	Generalized	10	26	16	(+)	NA	(+)	NA	TM	IVMP, PLEX for severe relapse
Hironishi et al. (11)	1	F	Asian	Generalized	23	30	7	(+)	(−)	(+)	Hyperplasia	ON	AZA, oral steroids, intermittent IVMP, IVIG
Ikeda et al. (12)	1	F	Asian	NA	29	45	16	(−)	NA	(+)	Hyperplasia	TM	IVMP
Ikeguchi et al. (13)	3	2F, 1 M	Asian	Generalized	25, 41, 47	49, 53, 70	24, 12, 23	3(+)	2(+), 1(−)	2(+), 1(−)	Thymoma, 1Normal, 1NA	2TM, 1ON	IVMP, PLEX (in one case), oral steroids
Isbister et al. (14)	1	F	Asian	Generalized	28	36	8	(+)	NA	(+)	Hyperplasia	ON	NA
Jarius et al. (15)	10	10F	Caucasian	NA	11–33 (23.4)	20–67 (44.3)	0–46	9(+), 1(−)	10(+)	6(+), 4(−)	3Hyperplasia, 1Thymitis, 2Normal, 4NA	5TM, 5ON	NA
Kay et al. (16)	1	F	Asian	Ocular	44	49	5	(+)	(+)	(−)	NA	ON	AZA, oral steroids
Kister et al. (17)	4	4F	2African, 1Caucasian, 1Asian	2Ocular, 2 Generalized	38, 36, 17, 27	39, 41, 19, 38	1, 5, 2, 11	4(+)	2(+), 1(−), 1NA	4(+)	3Hyperplasia, 1NA	3ON, 1TM	AZA, steroids; IVMP+IVIG for relapses
Kohsaka et al. (18)	1	F	Asian	NA	53	60	7	(+)	(+)	(+)	Hyperplasia	TM	IVMP, IVIG, tacrolimus, steroids
Leite et al. (19)	16	15F, 1 M	11Caucasian, 3African, 1Asian, 1NA	2Ocular, 13 Generalized	12–47 (26.5)	23–67 (39.5)	−24-41	16(+)	16(+)	11(+), 5(−)	8Hyperplasia, 3Normal, 5NA	8ON, 8TM	AZA + steroids, rituximab, cyclophosphamide
Nakamura et al. (20)	1	F	Asian	NA	28	38	10	(+)	(+)	(+)	NA	ON	IVMP
O’Riordan et al. (21)	1	F	Caucasian	NA	NA	41	NA	(+)	NA	NA	NA	ON	IVMP, cyclophosphamide
Ogaki et al. (22)	1	F	Asian	Generalized	30	43	13	(+)	(+)	(+)	NA	ON	IVMP, oral steroids
Spillane et al. (23)	1	F	NA	Generalized	23	31	7	(+)	(+)	(+)	Normal	TM	AZA, oral steroids
Tsujii et al. (24)	1	F	Asian	Generalized	10	33	23	(+)	(+)	(+)	Hyperplasia	ON	IVMP (ineffective), PLEX, oral steroids
Uzawa et al. (25)	2	2F	Asian	Generalized	20, 18	41, 27	21, 9	2(+)	2(+)	2(+)	1Hyperplasia, 1NA	2ON	IVMP, PLEX, oral steroids, tacrolimus
Vaknin et al. (26)	15	10F, 5 M	NA	NA	NA	NA	NA	11(+), 4(−)	7(+), 8(−)	9(+), 6(−)	7Thymoma, 2Hyperplasia, 6NA	14ON, 1TM	NA
Vakrakou et al. (27)	2	2F	Caucasian	Generalized	24, 17	49, 21	25, 4	2(+)	2(+)	2(+)	Normal	1TM, 1ON	IVMP, PLEX, AZA, oral steroids, rituximab (in one case)
Yau et al. (28)	1	F	Asian	Ocular	56	51	−5	(+)	(+)	(−)	NA	ON	IVMP, AZA, oral steroids
This case	1	F	Asian	Ocular	38	43	5	(+)	(+)	(−)	NA	TM	IVMP, eculizumab

MG, myasthenia gravis; NMOSD, neuromyelitis optica spectrum disorder; AQP4-Ab, aquaporin-4 antibody; AChR-Ab, acetylcholine receptor antibody; ON, optic neuritis; TM, transverse myelitis; IVMP: Intravenous Methylprednisolone; PLEX: Plasma Exchange; AZA: Azathioprine; IVIG: Intravenous Immunoglobulin; NA: Not Available.

Serologically, the vast majority of patients were positive for disease-specific autoantibodies: AChR-Ab was positive in 90.5% of tested patients, and AQP4-Ab was positive in 73.0% (Figure 2E). A high proportion of patients (70.3%) had undergone thymectomy. Among those with reported histopathology (*n* = 41), thymic hyperplasia was the most common finding (53.7%), followed by thymoma (22.0%) and normal histology (22.0%).

The temporal relationship between the two disorders was prominent. In the majority of cases (93.2%, 69/74), MG onset consistently preceded NMOSD onset by years (mean interval 12.56 years). The mean age of onset for MG was 28.19 ± 11.78 years, which was significantly younger than the mean age of NMOSD onset at 41.02 ± 13.40 years (*p* < 0.001). The interval between diagnoses varied widely, with a mean interval of 12.56 ± 12.99 years. The distribution of these intervals is visualized in Figure 2F.

## 4 Discussion

The sequential occurrence of MG and NMOSD is uncommon, and their overlapping epidemiological characteristics remain inadequately defined. MG has an estimated annual incidence of 1.5–3.1 per 100,000, with onset possible across all ages but more common in women aged 20–50 years and men over 65 (29, 30). In contrast, NMOSD shows a lower incidence of about 0.278 per 100,000 and predominately affects young to middle-aged women (31, 32). Consistent with known female preponderance in autoimmunity, our review identified a marked female predominance (89.2%) among patients with both conditions. The cohort was ethnically diverse, with Caucasians (36.5%) and Asians (25.7%) representing the majority. Although MG generally preceded NMOSD, the sequence of onset occasionally varied, as observed in some reports and in the present case.

Both MG and NMOSD are antibody-mediated autoimmune disorders, but they differ in target tissues and primary immunopathological mechanisms. MG results from impaired neuromuscular transmission driven mainly by AChR-Ab, supported by cellular immunity and complement activation (1). In contrast, NMOSD is primarily humoral, with AQP4-Ab playing a key pathogenic role (2). Other immune-mediated disorders, such as autoimmune glial fibrillary acidic protein astrocytopathy, further illustrate the spectrum of CNS autoimmunity (33, 34). In our patient, positivity for both AChR-Ab and AQP4-Ab reinforces the contribution of disease-specific autoantibodies. Notably, MG symptoms did not recur or worsen after NMOSD onset, possibly reflecting immune rebalancing. The frequent co-occurrence of both antibodies and the observed temporal pattern suggest a shared immunological predisposition, as also indicated by studies on the causal links between NMOSD and other autoimmune diseases (35).

**Figure 3:**
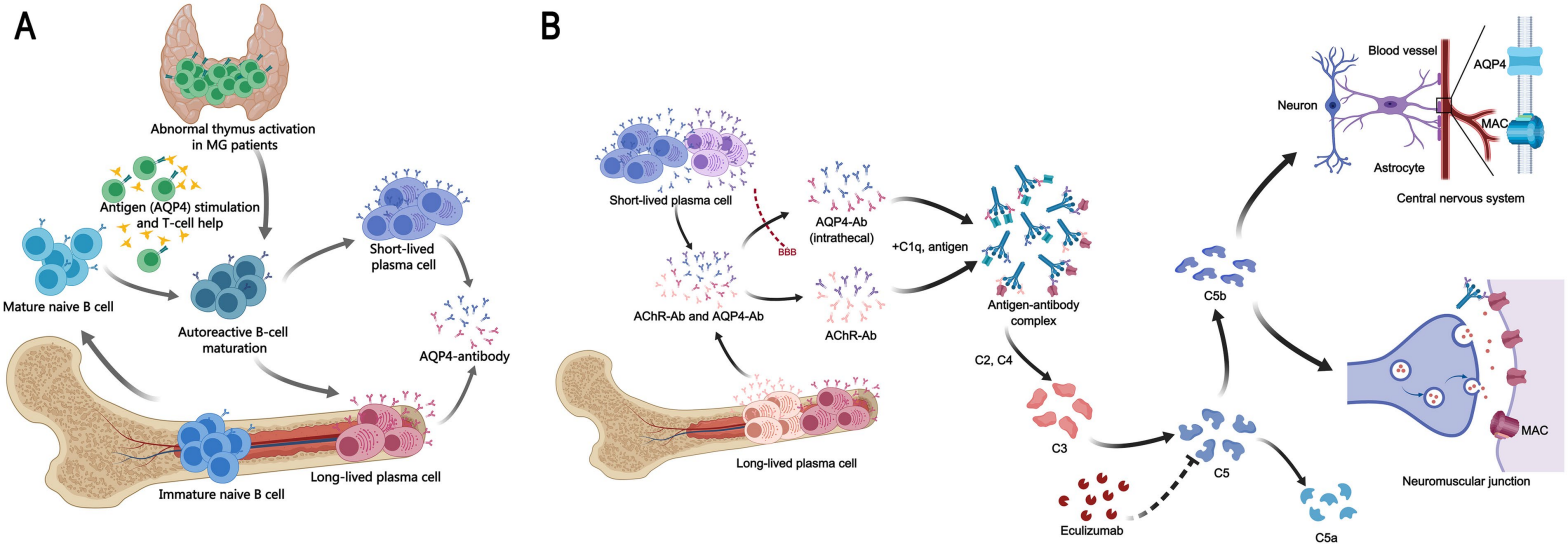
Mechanistic insights into the MG-NMOSD association and therapy. **(A)** Proposed immunopathogenic link between MG and NMOSD. A schematic illustrating a hypothesized cascade: MG, commonly with abnormal thymus activation like thymic hyperplasia, may induce systemic immune dysregulation. This environment could promote the emergence of autoreactive B cells that produce pathogenic AQP4-Ab, triggering NMOSD. This model may apply to a subset of patients, as NMOSD can develop post-thymectomy. **(B)** Mechanism of action of eculizumab in MG and NMOSD. The complement inhibitor eculizumab blocks cleavage of C5, preventing membrane attack complex (MAC) formation. In MG (below), this inhibits MAC-mediated damage at the neuromuscular junction initiated by AChR-Ab. In NMOSD (above), it blocks AQP4-ab-driven, complement-dependent astrocytic injury. Thus, it shares a molecular target (C5) but acts in distinct pathogenic contexts.

We propose a pathogenic model in which MG—often associated with thymic pathology—may initiate systemic B-cell dysregulation, eventually enabling the emergence of AQP4-specific autoimmunity (Figure 3A). This model accommodates cases where NMOSD develops even after thymectomy, indicating that autoreactive clones may become established independently of the thymic microenvironment. The discontinuation of mycophenolate mofetil prior to NMOSD onset in our case might have reduced immunosuppressive control, allowing breakthrough autoimmunity. Elevated complement C1q further supports complement dysregulation as a common pathway in both diseases, consistent with the recognized role of complement in their pathogenesis (34).

Management of patients with sequential MG and NMOSD remains clinically challenging. Early recognition of NMOSD in patients with established MG is crucial. Current treatment relies largely on immunomodulatory strategies overlapping between the two disorders, including steroids and conventional immunosuppressants. Plasma exchange and intravenous immunoglobulin may be used in acute severe attacks. However, therapeutic decisions must balance the severity and interaction of each disease. The development of targeted biologics offers promising alternatives. Agents such as rituximab, satralizumab, and eculizumab have shown efficacy in clinical studies. Updated 2024 NEMOS guidelines recommend eculizumab as a first-line therapy for AQP4-Ab-positive NMOSD due to its rapid onset and sustained C5 inhibition (36). Eculizumab is approved in China and several other countries for both AChR-Ab-positive refractory generalized MG and AQP4-Ab-positive NMOSD (37), supporting a shared complement-mediated mechanism. New therapeutic avenues potentially for MG-NMOSD overlap include FcRn inhibitors (e.g., efgartigimod), next-generation complement inhibitors (e.g., ravulizumab), and B-cell targeting agents (e.g., CD19 CAR-T). Personalized immunotherapy based on serological profiles may further optimize outcomes, while experimental approaches such as stem-cell or gene therapy warrant further investigation (1, 29, 36, 38).

In our patient, high-dose corticosteroids produced limited benefit, highlighting the refractory nature of her NMOSD presentation. The subsequent rapid response to eculizumab underscores the importance of complement activation and validates therapeutic targeting of this pathway. Eculizumab inhibits terminal complement component C5, thereby blocking the final effector mechanism in both AChR-Ab- and AQP4-Ab-mediated injury (Figure 3B). After three months of stable control with eculizumab, the patient was transitioned to a third-generation CD20 monoclonal antibody under a clinical trial protocol. This sequential approach—from complement inhibition to B-cell depletion—illustrates the dynamic and personalized management required for complex autoimmune overlap syndromes and introduces a promising new therapeutic option.

This study has several limitations. First, the analysis is based on published case reports, which are subject to publication bias (favoring severe/atypical presentations) and reporting bias, potentially inflating the observed female predominance and influencing the reported racial distribution. Second, diagnostic criteria evolved over time, and AQP4-IgG testing was not available for many historical cases, introducing heterogeneity and diagnostic uncertainty. Third, incomplete reporting of antibody status and clinical details, along with the lack of individual patient data, precluded advanced statistical analysis or meta-analysis. Finally, while a temporal association is clear, this study cannot establish causality or definitively prove a shared pathogenic mechanism between MG and NMOSD.

In conclusion, this analysis provides insight into the clinical characteristics and potential immunopathological links in patients with sequential MG and NMOSD. The consistent pattern of MG preceding NMOSD, the frequent presence of both AChR-Ab and AQP4-Ab, and the response to complement inhibition suggest overlapping autoimmune diatheses. Further large-scale, multicenter studies are needed to elucidate shared mechanisms and to develop more precise therapeutic strategies for this complex overlap syndrome.

## Data Availability

The original contributions presented in the study are included in the article/Supplementary material, further inquiries can be directed to the corresponding authors.
