# Supplementary material for: The temporal sequence of myasthenia gravis and neuromyelitis optica spectrum disorder: a case report and systematic review of 74 patients
Source: Front Neurol. 2026 Feb 13;17:1747855. doi: 10.3389/fneur.2026.1747855 (PMC12945749; doi:10.3389/fneur.2026.1747855)
Supplement: Supplementary file 1 [file Data_Sheet_1.PDF]

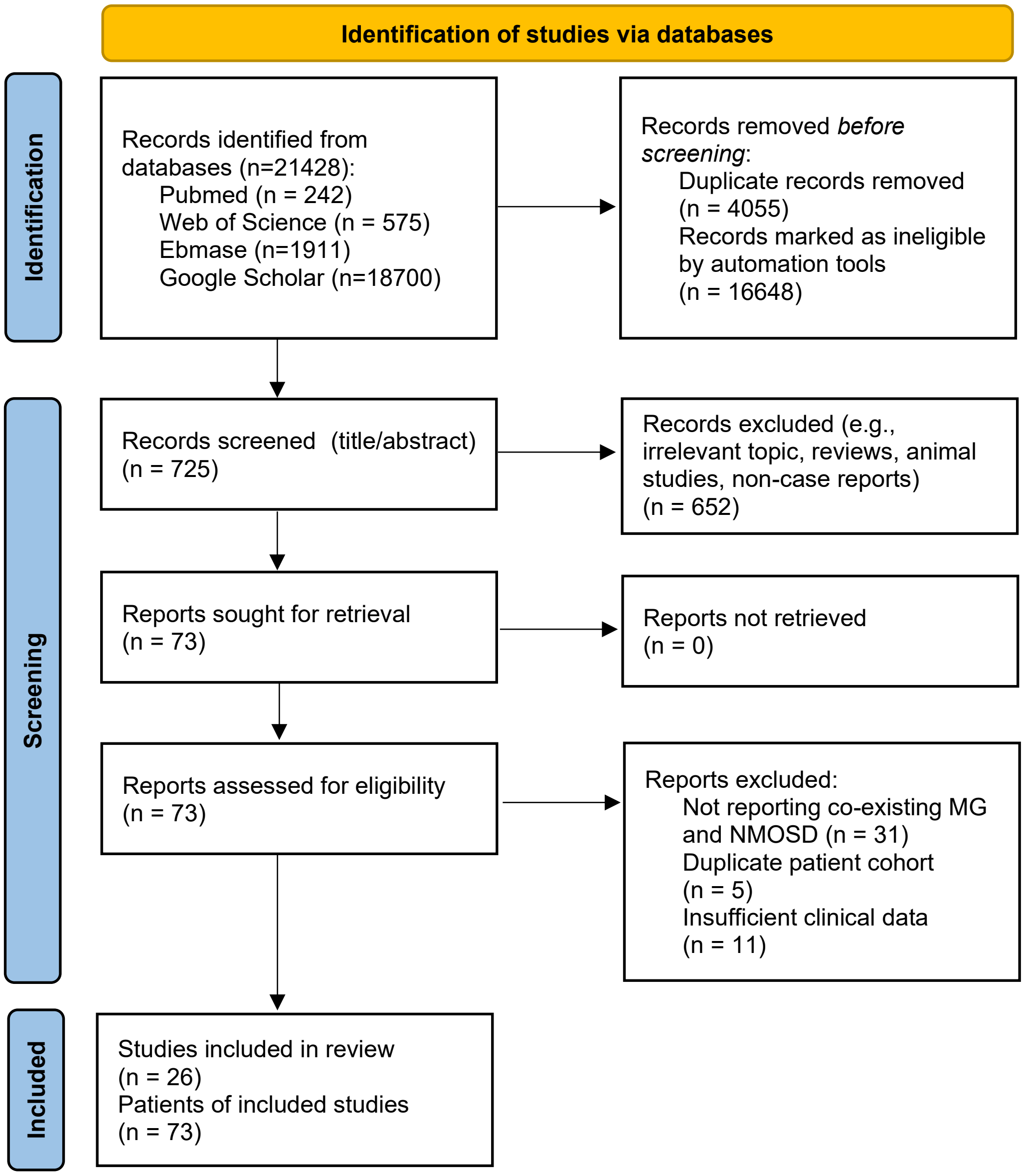


**Supplementary Figure S1. PRISMA Flow Diagram for Identification and Selection of Studies on Co-existing Myasthenia Gravis and Neuromyelitis Optica Spectrum Disorder**
